# Fluids in ARDS: more pros than cons

**DOI:** 10.1186/s40635-020-00319-x

**Published:** 2020-12-18

**Authors:** Renata de S. Mendes, Paolo Pelosi, Marcus J. Schultz, Patricia R. M. Rocco, Pedro L. Silva

**Affiliations:** 1 Laboratory of Pulmonary Investigation, Carlos Chagas Filho Biophysics Institute, Federal University of Rio de Janeiro, Centro de Ciências da Saúde, Avenida Carlos Chagas Filho, s/n, Bloco G-014, Ilha do Fundão, Rio de Janeiro, RJ 21941-902 Brazil; 2 Department of Surgical Sciences and Integrated Diagnostics (DISC), University of Genoa, Genoa, Italy; 3 San Martino Policlinico Hospital, IRCCS for Oncology and Neurosciences, Genoa, Italy; 4 Department of Intensive Care, Academic Medical Centre, University of Amsterdam, Amsterdam, the Netherlands; 5 Mahidol Oxford Tropical Medicine Research Unit (MORU), Mahidol University, Bangkok, Thailand; 6 Nuffield Department of Medicine, University of Oxford, Oxford, UK

**Keywords:** Acute respiratory distress syndrome, Balanced solution, Non-balanced solutions, Colloids, Human albumin, Hemodynamic

## Abstract

In acute respiratory distress syndrome (ARDS), increased pulmonary vascular permeability makes the lung vulnerable to edema. The use of conservative as compared to liberal fluid strategies may increase the number of ventilator-free days and survival, as well as reduce organ dysfunction. Monitoring the effects of fluid administration is of the utmost importance; dynamic indexes, such as stroke volume and pulse pressure variations, outperform static ones, such as the central venous pressure. The passive leg raise and end-expiratory occlusion tests are recommended for guiding fluid management decisions. The type of intravenous fluids should also be taken into consideration: crystalloids, colloids, and human albumin have all been used for fluid resuscitation. Recent studies have also shown differences in outcome between balanced and non-balanced intravenous solutions. In preclinical studies, infusion of albumin promotes maintenance of the glycocalyx layer, reduces inflammation, and improves alveolar-capillary membrane permeability. Fluids in ARDS must be administered cautiously, considering hemodynamic and perfusion status, oncotic and hydrostatic pressures, ARDS severity, fluid type, volume and infusion rate, and cardiac and renal function. Of note, no guideline to date has recommended a specific fluid composition for use in ARDS; most physicians currently follow recommendations for sepsis.

## Background

Acute respiratory distress syndrome (ARDS) is a multifactorial syndrome caused by different etiologies, which can be pulmonary or extrapulmonary [1]. A major feature of ARDS is increased pulmonary vascular permeability [2]; consequently, interstitial and alveolar edema are hallmarks of its pathophysiology. In addition, lung inflammation, damage to epithelial and endothelial cells, disruption of the extracellular matrix [3], and coagulation disturbances [4] have been recognized.

Mitigating lung edema, accelerating its resorption, and maintaining systemic perfusion and distal organ function through modulation of fluid intake seem to be beneficial in ARDS. Several issues should be considered during fluid infusion in this patient population: (1) the etiology, severity, and phase of ARDS; (2) static and dynamic methods to monitor fluid administration; (3) the type of fluids (crystalloids versus colloids, balanced versus non-balanced solution); and, (4) if albumin is used, its concentration. The present review will discuss recent studies on different fluid types and strategies and their effects on the aforementioned aspects of ARDS.

## Importance of ARDS pathophysiology when choosing the amount and type of fluids

Early ARDS is characterized by increased alveolar-capillary barrier damage, which results in lung edema, as well as reduced peripheral perfusion [4]. Increased lung water content impairs lung mechanics and gas exchange, leading to hypoxemia and increased risk of pulmonary arterial hypertension. Moreover, the presence of lung edema perpetuates lung inflammation and epithelial cell damage, thus reducing surfactant production and hindering edema reabsorption [4, 5].

Choosing the optimal fluid strategy (both regarding quantity and type of fluid) is one of the greatest clinical challenges in ARDS management, since both impaired hemodynamics and electrolyte disturbances must be corrected [6, 7], but either one may exacerbate pulmonary edema.

**Fig. 1:**
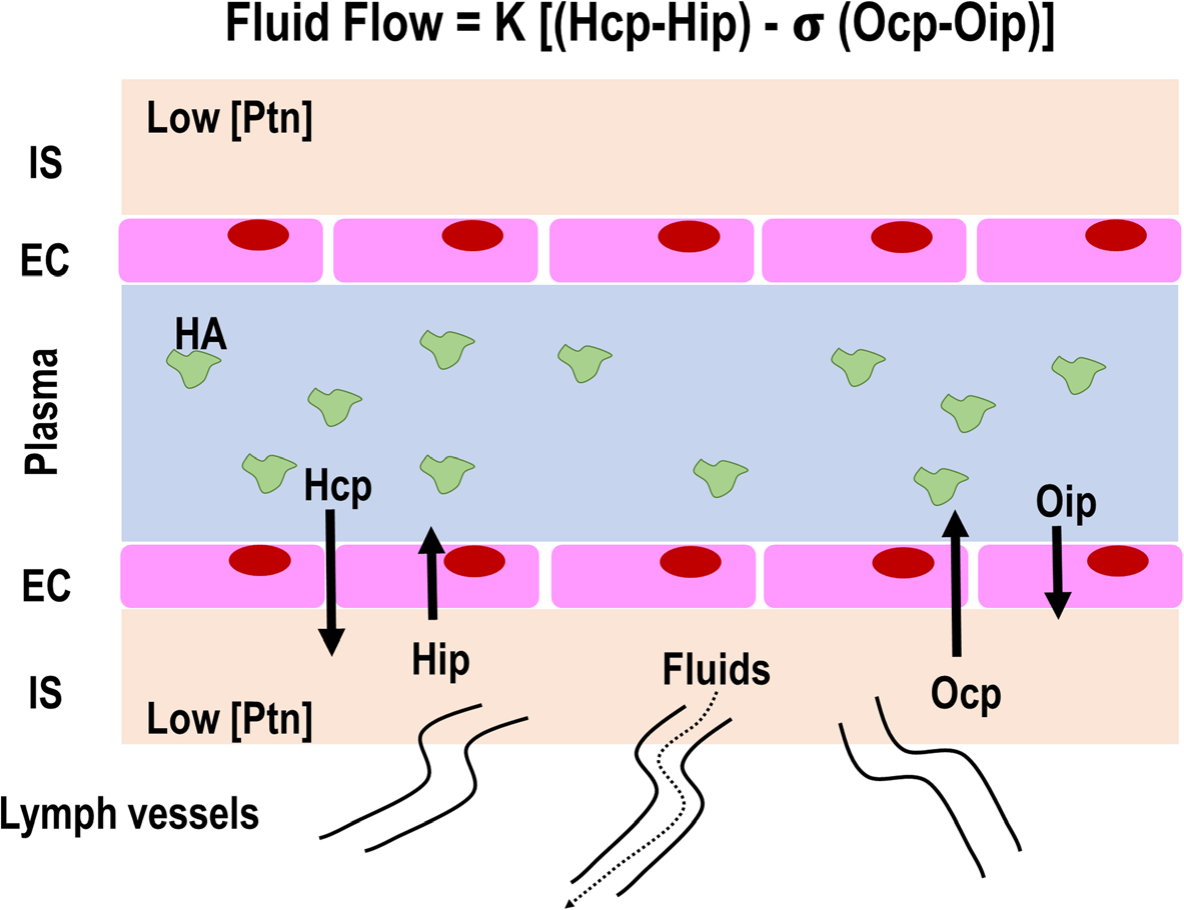
Fluid filtration according to the classic Starling model. Fluid filtration is mainly guided by the difference between the hydrostatic gradient [hydrostatic capillary pressure (Hcp) and hydrostatic interstitial pressure (Hip) multiplied by *K* (permeability index)] and oncotic gradient [oncotic capillary pressure (Ocp) and oncotic interstitial pressure (Oip) multiplied by *δ* (reflection coefficient)]. The interstitial space (IS) is considered to have a low protein concentration [Ptn]. Residual fluids situated in the IS would return to the plasma compartment guided mainly by Ocp; little fluid would return through the lymph vessels

**Fig. 2:**
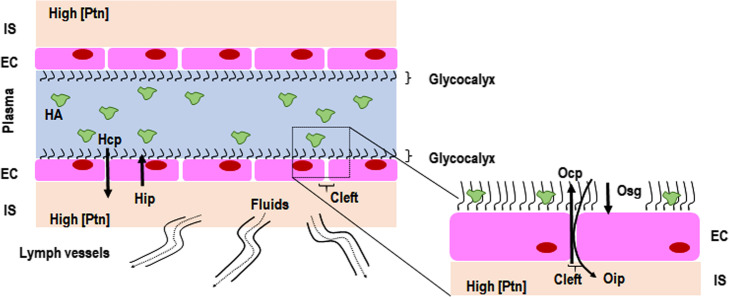
New fluid filtration model. This model proposes that the glycocalyx, which is composed of a glycoprotein skeleton and proteoglycans, interacts with circulating cells and plasma constituents (including albumin) to form an endothelial surface up to 1 μm thick. The interstitial space is considered to have a high protein concentration (Ptn) and, thus, a high oncotic interstitial pressure (Oip), which decreases fluid return to the plasma compartment. Additionally, due to the retention of human albumin molecules in the glycocalyx layer, this structure may generate its own oncotic pressure (Osg), which further jeopardizes fluid return to the plasma compartment. The residual fluid volume in the IS in this model is much greater than in the classic Starling model, and excess fluid may return through the lymph vessels

Starling’s classic model states that transvascular fluid exchange depends on a balance between hydrostatic and oncotic pressure gradients (Fig. 1) [8]. However, this theory has been considered inconsistent with our new understanding of the role of the glycocalyx [9]. It is now known that the interstitial space has a high protein concentration [8], which weakens the concept of fluid flow direction being guided solely by the difference in capillary and interstitial oncotic pressures. On the other hand, the subglycocalyx space is also responsible for oncotic pressure gradients (Osg) that may determine the transcapillary flow. The functional barrier created by the endothelial glycocalyx layer—which is composed of a glycoprotein skeleton, proteoglycans, and interactions with circulating cells and plasma constituents, including albumin—forms an endothelial surface up to 1 μm thick [8]. The luminal side of the endothelium, below this “protein sponge,” is permanently removed through small breaks (clefts) in the intercellular junction chains to adjacent tissues [10], thus creating an inwardly directed oncotic force (Oip), quantitatively opposed to hydrostatically (Hcp) driven fluid filtration (Fig. 2). Lymph vessels are responsible for the return of fluid to the circulation, which is regulated through sympathetically mediated responses [8]. Although albumin infusion may improve or maintain vascular barrier competence, experiments with isolated organs show that the surface functions well until the albumin concentration falls below 10 g/L [11]. Therefore, it is very likely that the major insult to the vascular barrier leading to its dysfunction is not hypoalbuminemia, but damage to the endothelial glycocalyx. Several factors are associated with glycocalyx damage, such as hypervolemia [12], rapid crystalloid infusion [13], ischemia-reperfusion [14], inflammation [8], sepsis [15], hyperglycemia [16], trauma [17], and altered plasma pH [18]. The glycocalyx is the first and the primary source of resistance to the stream of fluid and solutes between plasma and lymph. Compaction of endothelial glycocalyx and the consequent increase in levels of glycosaminoglycans (GAGs—heparan, hyaluronic acid, or chondroitin) in plasma are considered markers of glycocalyx injury [19, 20]. In this context, circulating levels of syndecan-1 and GAGs have been found elevated in septic patients [15, 21, 22] and are associated with disease severity and worse outcomes [21].

## Fluid volume and monitoring in ARDS patients

Conservative fluid replacement strategies have been recommended to prevent lung edema. These strategies are associated with less invasive mechanical ventilation, less organ dysfunction, and a trend toward less renal replacement therapy [6, 23]. A recent systematic review and meta-analysis showed that conservative or deresuscitative fluid strategies resulted in an increased number of ventilator-free days and a decreased length of ICU stay compared with a liberal strategy or standard care in adults and children with ARDS, sepsis, or systemic inflammatory response syndrome. Nevertheless, the effect on mortality remains uncertain [6]. In an attempt to better identify those patients who would benefit from fluid replacement, aldosterone and B-type natriuretic peptide (BNP) have been assessed as possible outcome predictors. The authors found that lower levels of aldosterone seem to identify ARDS patients for whom conservative fluid management may improve mortality, while BNP levels did not predict outcomes [24]. In a recent study, conservative fluid management was found to improve mortality in black patients with ARDS [25], suggesting that fluid strategies may be personalized in the future.

## Hemodynamic monitoring

To date, no ideal method has been developed to continuously monitor the response to fluid replacement in ARDS. Four questions have been described to guide fluid strategy in patients with septic shock: “When to start intravenous fluids?”, “When to stop intravenous fluids?”, “When to start de-resuscitation or active fluid removal?”, and, finally, “When to stop de-resuscitation?” [7]. These questions are also pertinent when dealing with non-sepsis states [7]. Several tests and devices are available to predict fluid responsiveness [26]. Static markers of cardiac preload, such as central venous pressure (CVP), do not reliably predict fluid responsiveness; dynamic indexes should be used instead [27].

## Dynamic indexes to predict fluid responsiveness

### The passive leg raise test

The passive leg raise test (PLR) [28] consists of moving a patient from the semi-recumbent position to a position where the legs are lifted at 45° and the trunk is horizontal. The transfer of venous blood from the lower limbs and the splanchnic compartment toward the cardiac cavities mimics the increase in cardiac preload induced by a fluid bolus of roughly 300 ml [28]. This test is able to predict fluid responsiveness [29]; the threshold to define responsiveness is a 10% increase in stroke volume and/or cardiac output (CO) after PLR.

### Stroke volume variation (SVV) and pulse pressure variation (PPV)

SVV and PPV, derived from the arterial pressure signal, represent the influence of the periodic increase in intrathoracic pressure due to invasive mechanical ventilation. For SVV and PPV to be accurate predictors of fluid responsiveness, at least four conditions are necessary: sedation and paralysis, tidal volume (*V*_T_) ≥ 8 ml/kg, regular heart rhythm, and respiratory system compliance ≥ 30 ml/cm H_2_O [30, 31]. Several studies have shown that an SVV greater than 10% or a PPV greater than 13–15% are predictive of fluid responsiveness [32].

### The end-expiratory occlusion (EEO) test

The EEO test is easy to perform in patients under invasive mechanical ventilation [33]. Ventilation is briefly interrupted (15–30 s), which increases the right cardiac preload to its maximum value. Depending on the duration of occlusion, transmission occurs to the left side. An increase in stroke volume is indicative of preload responsiveness [33]. The EEO test proved reliable in several settings [34, 35], especially when *V*_T_ ≥ 8 ml/kg, but not with *V*_T_ = 6 ml/kg [36]. Therefore, caution is warranted in the use of the test, since a protective tidal volume is always recommended in ARDS. EEO may predict fluid responsiveness even in prone position and during the Trendelenburg maneuver [37].

## Types of fluids in ARDS

It is well established that, due to the potential impact on alveolar-capillary membrane damage and lung edema, the type of fluid infused may influence ARDS outcome [38, 39]. However, there is no consensus to date regarding the best fluid for use in ARDS.

## Crystalloids versus colloids

The ALBIOS trial study evaluated 1818 critically ill patients and randomized them to two types of replacement fluid: 20% albumin plus crystalloid or crystalloid only. In the intervention group, the goal of therapy was to achieve plasma albumin levels > 30 g/L. There was no difference in overall mortality at 28 days. An ongoing trial (NCT03869385) was designed to assess the impact of 20% albumin with crystalloid with a target albumin level of > 30 g/L in patients with septic shock, with 28-day mortality as the primary endpoint. Another ongoing clinical trial (NCT03654001) is comparing 20% albumin plus balanced solutions, 20% albumin plus saline, and balanced solutions alone in septic shock. No clinical trial has evaluated albumin or other types of fluids specifically in ARDS, and none is ongoing.

## Balanced versus non-balanced

Crystalloids can also be categorized as balanced or non-balanced (unbalanced) solutions. Balanced solutions contain a precursor anion of bicarbonate in their composition: lactate in Ringer’s lactate, acetate in Ringer’s acetate and Plasma-Lyte®, and others such as gluconate, malate, citrate, and succinate in less commonly used solutions [40]. Non-balanced solutions (such as 0.9% NaCl) are unbuffered and have higher chloride content.

Balanced solutions provide some advantages, not least the choice of anion: they (1) replace bicarbonate, (2) are rapidly metabolized, (3) are nontoxic, and (4) maintain pH at 4–8, minimizing hemolysis and endothelial cell damage in peripheral circulation [40]. However, some issues unique to each of the different anions used in balanced solutions must be taken into account before choosing one. For example, almost 70% of infused lactate will participate in gluconeogenesis, being converted to pyruvate and potentially destabilizing glycemic control [41]. Pyruvate follows the Krebs cycle via acetyl-coA, generating carbon dioxide. If lactate accumulates, glycolysis is delayed at the glyceraldehyde-3-phosphate stage, thus reducing ATP generation [41]. On the other hand, acetate has certain theoretical advantages over lactate, as it is metabolized more rapidly (300 mmol/h) and completely (i.e., without accumulating). Acetate does not lead to hyperglycemia and has less effect on oxygen consumption and carbon dioxide elimination. Furthermore, unlike lactate, acetate is metabolized in various non-hepatic tissues, particularly muscle, so is less subject to accumulation in shock or liver dysfunction. The half-life of unbalanced solutions is longer than that of balanced solutions [42], which should be considered when choosing fluid type in different clinical scenarios. Finally, the higher chloride concentration of unbalanced solutions leads to reduced renal flow and increased renal injury [43, 44].

However, clinical studies comparing balanced versus unbalanced crystalloids in intensive care have shown conflicting results regarding renal outcomes and mortality [44–48]. The SPLIT study randomized 2278 patients to receive Plasma-Lyte® versus 0.9% saline [47]. There was no difference in the proportion of patients with acute, moderate, or severe renal injury. (Nevertheless, it bears stressing that the SPLIT study enrolled patients at low risk of acute kidney injury and that the average volume of fluid infused was less than 2000 mL). The SMART study randomized 15,802 patients to receive 0.9% saline versus balanced crystalloid (Ringer lactate or Plasma-Lyte®) in five intensive care units [49]. The authors showed an association between reduced risk of major renal effects (need for renal replacement therapy, death and/or creatinine at endpoint > 200% at admission) and the use of balanced solutions. In an emergency department setting, the SALT-ED study included non-critically ill patients and compared 0.9% saline versus ringer lactate or Plasma-Lyte® [50]. Although mortality at 28 days did not differ between groups, fewer renal adverse events were observed in the group receiving balanced solutions.

## Colloids

Colloids are water-based solutions containing permeable ions and non-permeable molecules, which can be plasma-derived (albumin) or synthetic (gelatins, starches, and dextran) and are unable to cross intact semipermeable biological membranes. The intravascular duration of colloid-derived volume expansion depends on the rate of metabolism and clearance of its constituent molecules [51]. Colloid turnover is described according to the elimination half-life of the oncotic macromolecules contained in the fluid. The half-life of crystalloids is usually expressed in minutes, while elimination of colloids can take hours [42]. Importantly for clinical practice, these elimination half-lives do not reflect the duration of plasma expansion, which is much shorter. This means that macromolecules persist for many hours outside the bloodstream, impregnating renal tissue and thus promoting osmotic nephrotic injury [52], urinary hyperviscosity (which causes tubular obstruction), and inflammation [53]. Synthetic colloids have a well-established association with severe adverse events [54]; human albumin remains under clinical evaluation in this respect.

## Albumin in ARDS

Human albumin is commercially available in several concentrations: iso-oncotic (5%) and hyper-oncotic (20% and 25%) [55]. However, few studies have compared these concentrations in terms of impact on clinical outcomes.

Albumin has many other properties in addition to its effects on intravascular volume, including antioxidant activities and molecular transport [55]. Clinical research has shown improvement in physiological parameters after human albumin administration with or without furosemide in patients with ARDS [56]. The antioxidant effect of albumin is related to its capacity to bind iron and copper, thus reducing the availability of these components for pro-oxidant reactions [57]. Changes in albumin concentration and structure during critical illness can lead to significant changes in homeostasis, metabolism, and drug distribution and efficacy [57]. Antimicrobial binding capacity has been shown to be decreased in patients with hypoalbuminemia [58]. These changes may result in inadequate treatment, particularly for time-dependent antibiotics, which may require dose adjustments [58]. Albumin can also protect the microvasculature and attenuate increased vascular permeability by reducing capillary bed leakage [59, 60]. Finally, there is an inverse association of albumin concentration with levels of several pro-inflammatory cytokines [61].

**Fig. 3:**
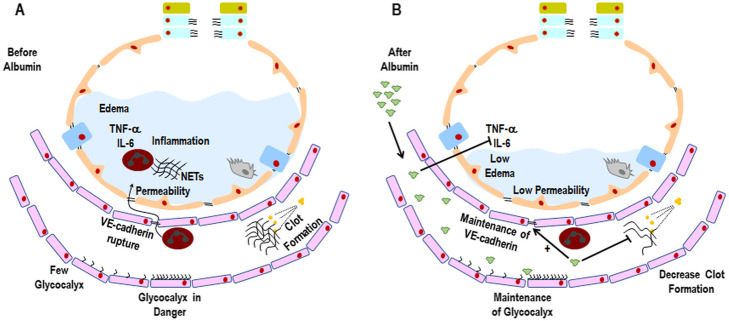
**a** *Before human albumin infusion.* In ARDS, glycocalyx content is reduced and its function may be impaired depending on the stage of disease. Due to increased levels of pro-inflammatory mediators in the alveolar compartment, neutrophils are chemoattracted to the alveolar space, trespassing and causing damage to both endothelial cell junctions (e.g., VE-cadherin) and epithelial cell junctions (e.g., E-cadherin). Therefore, alveolar-capillary membrane damage increases, thus contributing to interstitial and alveolar edema. Activation of neutrophils in the alveolar space may promote the release of elastase and the production of neutrophil extracellular traps (NETs). Clot formation is increased, leading to aggregation of platelets into fibrin nets, which impair ventilation-perfusion ratio and decrease oxygenation. **b** *After human albumin infusion.* Human albumin is able to reduce levels of pro-inflammatory mediators in the alveolar compartment. Theoretically, this would reduce neutrophil attraction to the alveoli, thus reducing endothelial and epithelial cell damage and leading to decreased alveolar-capillary membrane permeability and lung edema. Human albumin is also able to maintain the glycocalyx connected to the luminal surface of endothelial cells. Finally, due to its heparin-like molecular structure, human albumin is able to reduce clot formation, which might improve ventilation-perfusion ratio and oxygenation

Lung tissue endothelial VE-cadherin expression is reduced in ARDS [3]. In vitro studies have reported rupture of endothelial adherens junctions, corroborating this clinical finding [62]. Reduction of VE-cadherin expression has been associated with increased vascular permeability in ARDS [3] (Fig. 3a). In experimental ARDS, both iso- (4%) and hyper-oncotic (20%) human albumin preserved the integrity of the endothelial barrier compared to Ringer lactate [63], in a mechanism apparently mediated by reduced lung inflammation [64].

Activation of endothelial cells (by microorganisms, toxins, or ischemia-reperfusion injury) leads to the production of mediators (such as angiopoietin-2) and neutrophil infiltration, thus increasing lung vascular permeability. Neutrophil-mediated epithelial injury has been associated with multiple mechanisms: tumor necrosis factor (TNF)-α, neutrophil extracellular traps (NETs), and TNF-related apoptosis-inducing ligand (TRAIL) [65, 66]. In preclinical ARDS studies, albumin attenuated lung damage by reducing oxidative stress and pulmonary inflammation [38, 39, 63]. However, in vitro [67] and in vivo [68] studies reported that hyper-oncotic albumin (20–25%) was associated with renal dysfunction proportional to its concentration. Accordingly, in endotoxin-induced ARDS, iso-oncotic albumin (4%) reduced both lung and kidney damage [63]. A meta-analysis found that albumin improved oxygenation but did not affect mortality in ARDS [69].

Activation of the coagulation cascade is also involved in the pathophysiology of ARDS [70]. Albumin has the ability to inhibit platelet aggregation in a heparin-like manner, albeit with lower potency [71], due to structural similarities between the two. Therefore, in theory, albumin replacement may attenuate the coagulation cascade in ARDS when administered at the right time and dose. In critically ill patients, albumin administration resulted in prolonged activated partial thromboplastin time (APTT) [72]. In vitro studies using thromboelastometry measurements reported impaired clot formation in the hyper-oncotic compared to iso-oncotic albumin group [73]. The hypoalbuminemia often presents in cancer and critical illness may contribute to venous thromboembolism [73].

Albumin reduces lung edema and restores/maintains glycocalyx integrity in experimental ARDS [63]. Perhaps accordingly, iso-oncotic and hyper-oncotic albumin were associated with less lung edema compared with Ringer lactate in the same study [63]. Furthermore, albumin reduced glycocalyx damage in experimental models of ARDS induced by ischemia-reperfusion [8, 74, 75] and hemorrhagic shock [76] (Fig. 3b).

## Conclusion

ARDS is characterized by lung edema and damage to alveolar epithelium, endothelium, and extracellular matrix. Fluid administration is thus very challenging in these patients, since it can increase cardiac output and peripheral perfusion—thus improving tissue oxygenation and organ function—but may also result in serious adverse events. Fluid overload may be particularly dangerous in ARDS due to the possibility of worsening pulmonary edema.

Different methods have been used to assess the appropriateness of fluid infusion. However, clinical parameters frequently employed at bedside, such as mean arterial pressure, urine output, oxygen consumption, lactate, and central/mixed venous oxygen saturation, are actually of little use. Static monitoring parameters (e.g., central venous pressure) are unreliable markers of fluid responsiveness; thus, dynamic parameters (PLR, SVV, PPV, and EEO) have been recommended instead.

In short, fluids in ARDS must be administered cautiously, considering hemodynamic and perfusion status, oncotic and hydrostatic pressures, ARDS severity, fluid type, volume and infusion rate, and cardiac and renal function. To date, there is no published evidence to recommend any specific fluid composition over another in ARDS, and most physicians follow recommendations designed for sepsis instead. Further research is needed to help elucidate this issue with a view to improving clinical management of ARDS.

## Data Availability

Not applicable

## Abbreviations

ARDS: Acute respiratory distress syndrome
APTT: Activated partial thromboplastin time
BNP: B-type natriuretic peptide
CO: Cardiac output
CVP: Central venous pressure
EEO: End-expiratory occlusion test
GAGs: Glycosaminoglycans
Hcp: Hydrostatic capillary pressure
Hip: Hydrostatic interstitial pressure
IS: Interstitial space
NET: Neutrophil extracellular trap
Osg: Oncotic pressure
Oip: Oncotic interstitial pressure
Ocp: Oncotic capillary pressure
PLR: Passive leg raise test
PPV: Pulse pressure variation
SVV: Stroke volume variation
TNF-α: Tumor necrosis factor alpha
TRAIL: TNF-related apoptosis-inducing ligand
*V*_T_: Tidal volume
